# Optimizing Printhead Design for Enhanced Temperature Control in Extrusion-Based Bioprinting

**DOI:** 10.3390/mi15080943

**Published:** 2024-07-24

**Authors:** Ruhan Guo, Wencheng Tang

**Affiliations:** School of Mechanical Engineering, Southeast University, Nanjing 211189, China; 230198918@seu.edu.cn

**Keywords:** bioprinting, bioink, finite element analysis

## Abstract

This study addresses critical challenges in the field of tissue engineering, specifically in the optimization of bioprinting technologies for the construction of complex, multicellular tissues. By utilizing a homemade piston-driven extrusion-based bioprinting (EBB) printhead, we performed detailed thermal and flow analyses to investigate the effects of temperature variations on the extrusion process of temperature-sensitive gelatin-alginate bioink. Through finite element method (FEM) simulations, we explored the temperature distribution within the printhead and its impact on bioink properties, such as viscosity, pressure, and shear stress. Key findings reveal significant temperature gradients from the printhead barrel to the nozzle tip, influencing bioink extrusion and filament morphology. This study further introduces an innovative hardware optimization with thermal insulators, designed to mitigate heat loss at the nozzle tip and ensure uniform temperature distribution. Both simulation and empirical printing experiments confirm the efficacy of thermal insulators in enhancing bioprinting fidelity and efficiency. This research contributes to the advancement of bioprinting technology by optimizing printhead design, with implications for improving the quality of bioprinted tissues and organs.

## 1. Introduction

Tissue engineering is a promising tool to solve the issue of organ shortage due to supply–demand imbalance, and the goal is to assemble functional constructs that restore, maintain, or improve damaged tissues or whole organs [1,2]. Nevertheless, traditional tissue engineering strategies encounter challenges in the construction of multicellular complex tissues and even personalized structures [3]. Bioprinting is an advanced tissue engineering approach, which utilizes cells, proteins, and biomaterials as raw materials, i.e., bioink, for the additive manufacturing of biological products [4,5,6].

The major challenge faced by bioprinting technology is how to dispense bioink into discrete units through a material dispensing system (usually called a printhead). Therefore, bioprinting is normally categorized by dispensing technology. Extrusion-based bioprinting (EBB) is one of the most popular bioprinting technologies currently available, where bioink located in a reservoir is pushed by a driving mechanism and extruded as continuous filaments via a printhead nozzle [7]. EBB technology is widely used mainly because it is compatible with most bioinks with viscosities ranging from 30 mPa·s to 6 × 10^7^ mPa·s at a reasonable cost [8].

In general, there are three different driving mechanisms of EBB printheads: piston-driven, pneumatic-driven, and leadscrew-driven. Since a leadscrew-driven printhead usually has relatively lower cell viability due to the larger pressure drop inside reservoir [9], piston- and pneumatic-driven printheads are commonly used for higher cell viability (90%) [7,8]. Theoretically, a piston-driven printhead has even better printing precision because the output flow volume of bioink is dependent on the feed rate of the piston [7,10]. In contrast, in practice, trial-and-error methods are used to achieve high precision and reproductivity [11], since the control is more complex than a pneumatic-driven printhead. One of the major reasons here is the non-Newtonian behavior of bioink, which leads to complexity. The ideal material properties of bioink are high mechanical strength with shear-thinning behavior, as well as great biocompatibility, which ensure an excellent balance of printability, structural support, and cell proliferation [12,13]. To be specific, to achieve higher printing resolution, bioink needs to improve its viscoelasticity to maintain the mechanical properties of the layer-by-layer structure. However, by doing this, embedded cells will experience an increase due to higher shear stress during the printing process, resulting in poor cell viability. On the other hand, bioink with lower viscosity will protect resident cells from shear stress during extrusion, but it will have unsatisfied structural fidelity [14].

To solve the problem, large amounts of work have been carried out to develop novel bioink and crosslinking strategies. Guo developed a double-network hydrogel composed of hyaluronic acid and alginate, which featured both great injectability and mechanical strength for self-supporting bioprinting [15]. Ouyang introduced a new in situ crosslinking method to simultaneously crosslink the photo-sensitive hydrogels during extrusion [16]. Nevertheless, even with newly developed bioink, it will take a lot of effort to find out the proper parameters and factors required to achieve satisfactory printing results and cell viability, not to mention the fact that some of the bioinks are expensive and difficult to synthesize. Recently, the finite element method (FEM) has been used to study the printing process to help researchers gain a better understanding of the relationship among bioink properties, printing parameters, and printhead design. Juan applied the finite element method (FEM) to study the extrusion bioprinting process with different nozzle tip structures [17], as well as bioink temperature’s influence on shear stress, pressure, and velocity during bioprinting [18]. Hamed investigated the impacts of different velocities and viscosities on extrusion-based chitosan 3D printing [19]. While most of the simulation studies focused on investigating different printing parameters or specific materials, few of them thrived when optimizing bioprinter structure design based on simulation studies to adapt emerging materials.

In this study, we advance the field of tissue engineering by optimizing the design of a bioprinting printhead with enhanced temperature control, crucial for maintaining bioink integrity during printing. Our innovative approach integrates thermal and fluid dynamics analyses with FEM simulations, leading to a novel printhead design featuring thermal insulators that stabilize temperature-sensitive bioinks. This development not only improves the structural fidelity and cellular viability of printed tissues but also joins theoretical predictions with practical implementations, setting new benchmarks for the use of printhead technology in bioprinting. Our findings offer substantial advancements in bioprinting precision and efficiency, significantly contributing to regenerative medicine and organ fabrication.

## 2. Materials and Methods

### 2.1. Piston-Driven Extrusion-Based Bioprinting (EBB) Printhead

In this research, a custom-build piston-driven EBB printhead was designed and built for a bioprinting system. The printhead consisted of a linear motion system, a bioink loading unit, and a temperature-controlling system, which were mainly made of aluminum alloy. A stepper motor and leadscrew-based mechanism was used for the linear motion system, and the bioink loading unit was connected to the linear motion system. A 3 mL disposable syringe (Becton Dickinson, Franklin Lakes, NJ, USA) was designed to insert into the loading unit, and a pressing unit driven by the stepper motor was mounted to exert linear force on the thumb rest of the syringe plunger during printing. Additionally, a polyimide heater film (10 W) was adhered around a copper sleeve inside the printhead to serve as heat source for the printhead, and a temperature sensor (Pt100, Allymore, Shenzhen, China) was also installed to monitor the real-time temperature of the printhead.

### 2.2. Material Preparation and Rheological Analysis

Since both thermal and fluid analysis were performed in this study, a temperature-dependent material was used as the bioink. To prepare the material, UV-sterilized gelatin and alginate powders were evenly mixed and dissolved in 0.9% NaCl solution (m/v) at 10% and 1% (m/v), respectively. The solution was then sterilized by heating three times in an oven (70 °C) for 30 min.

Since the bioink used in this study exhibited shear-thinning behavior. Rotational and oscillatory tests were performed using an Anton Paar MCR 302 Rheometer (Anton Paar, Ostfildern, Germany) to investigate the rheological properties and the proper gel point. The viscosity parameters were measured at 15 °C, 30 °C, and 37 °C with a shear rate of 0.1–100 s^−1^. The measurement parameters of the oscillatory test used were a deformation range of 0.1%, a cone plate diameter of 25 mm, and a cone angle of 2°, and the distance between the cone tip and the base plate was 99 µm. The elasticity of the bioink was represented by the storage modulus G′, and the viscosity was represented by the loss modulus G″. G′ and G″ were calculated through a temperature sweep at a frequency of 1 Hz. Before measurement, all samples were heated in a water bath at 37 °C for 5 min to ensure that the material was in a sol state, with an initial temperature of 37 °C. The sol–gel temperature transition point test was completed through temperature scanning tests, with the samples being cooled from 37 °C to 20 °C at a rate of 1 °C per minute. Three parallel experiments were conducted on each experiment for reproductivity.

### 2.3. Cell Culture and Cell-Laden Structure Bioprinting

The Primary Human Umbilical Vein Endothelial Cells (HUVECs) were obtained from the Cell Resource Center, IBMS, CAMS/PUMC (Beijing, China) and cultured in high-glucose Dulbecco’s modified Eagle medium (H-DMEM; Invitrogen, Waltham, MA, USA) supplemented with 10% fetal bovine serum (FBS; Gibco, Waltham, MA, USA) under conditions of 37 °C and 5% CO_2_. The cells were passaged by 0.25% trypsin (Invitrogen) when the cell confluency reached about 90%. The culture media were changed every 2–3 days.

To prepare cell-laden bioink for bioprinting, the cells were first harvested by centrifugation at 1000 rpm for 5 min and suspended in cell culture medium to a density of 4 × 10^6^ cells/mL. Then, the HUVECs, 20% gelatin, and 4% sodium alginate were mixed at a volume ratio of 1:2:1 to obtain the bioink for bioprinting experiment. The final bioink consisted of 10% gelatin, 1% sodium alginate, and a cell density of 1 × 10^6^ cells/mL.

During bioprinting process, the bioink was loaded into a syringe to print a grid-like structure with geometric dimensions of 10 mm × 10 mm × 4 mm. After printing, the structure was crosslinked with 3% CaCl_2_ solution for 5 min. Following crosslinking, the structure was gently washed three times with phosphate-buffered saline (PBS) solution and then cultured in DMEM medium.

### 2.4. Cell Viability Analysis

In addition, printed cell-laden 3D structures were immediately assessed for cell survival conditions using a live/dead staining method. Specifically, the printed structures were washed three times with PBS, then incubated with a mixture of 1 μmol/L Calcein-AM (Dojindo, Kumamoto, Japan) and 2 μmol/L PI (Dojindo) at 37 °C in the dark room for 30 min. After incubation, the samples were gently washed three times with PBS and observed under a confocal microscope (C2^+^, Nikon, Tokyo, Japan). Three independent samples were set up as replicates, with three fields of view chosen randomly from each sample.

Cell proliferation was analyzed using the cell-counting kit-8 (CCK-8) assay kit (Dojindo) on days 1, 3 and 7. Specifically, each group of samples was washed three times with PBS, and then a mixture of 1 mL H-DMEM and 0.1 mL CCK-8 solution was added. The samples were incubated at 37 °C for 2 h. After incubation, the culture medium was transferred to a 96-well plate, and the optical density (OD) values were read at 450 nm using a microplate reader. Each group had three independent repeats at each time point.

### 2.5. Finite Element Analysis

**Figure 1:**
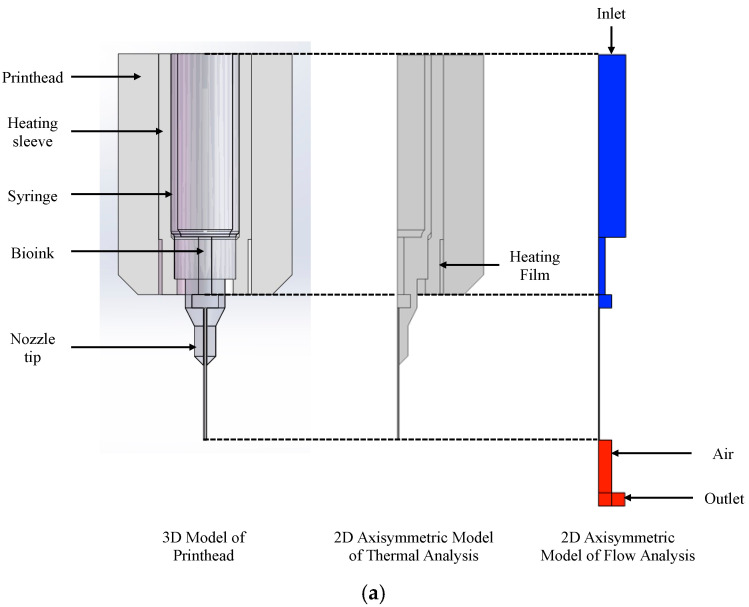

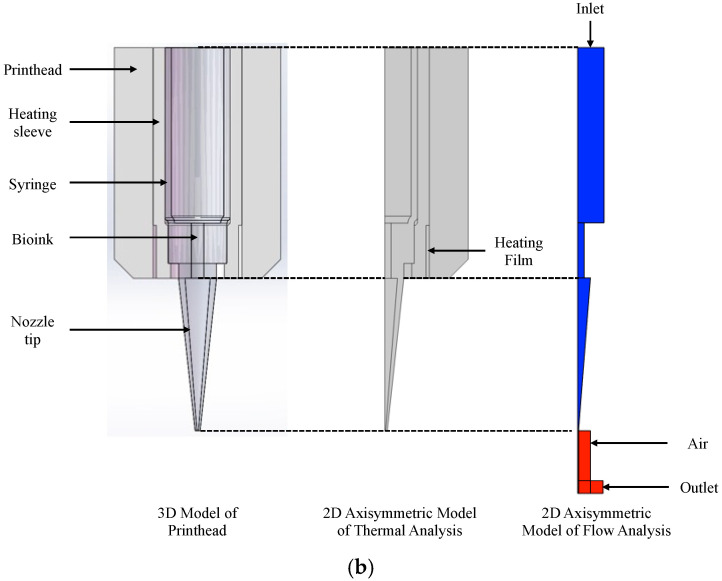
Three-dimensional and finite element analysis model: (**a**) model for printhead with stainless steel nozzle tip; (**b**) model for printhead with tapered nozzle tip.

The FE simulation was conducted in COMSOL Multiphysics 6.2 (COMSOL, Burlington, MA, USA) to study the temperature field of the printhead and the extrusion process of the hydrogel via the piston-driven printhead. To accelerate the simulation efficiency, simplified 2D axis-symmetric models were generated for both thermal and flow analysis based on 3D model of the lower-part printhead, as shown in Figure 1. Since each analysis focused on different target outcomes, the geometrical features were different, but both models shared the same critical geometries. To be specific, models of thermal analysis consisted of bioink, a syringe (with nozzle tip), a heating sleeve, and a printhead structure, while models of flow analysis consisted of bioink and air. The dimensions of syringe and nozzle were considered critical geometries for both simulations. In addition, two kinds of conventional nozzle tips (both were 250 µm), a 1/2-inch stainless steel (SS) tip (Figure 1a) and tapered tip (TT) (Figure 1b), were applied in the simulation study.

#### 2.5.1. Thermal Analysis of Printhead

Generally, heat transfer has three major mechanisms: thermal conduction, thermal convection, and thermal radiation. During bioprinting with gelatin-alginate bioink, the printhead was usually heated to 37 °C, which is a conventional heating temperature for gelatin-alginate based bioink with living cells. The bioink was around 4 °C before printing (as it was previously stored at fridge). The atmosphere temperature was 15 °C, which was the environment setup in the sterile cabinet at the biosafety laboratory. In this scenario, thermal conduction and convection play dominant roles in the temperature distribution of the printhead, while thermal radiation can be neglected.

Heat convection happened both inside and outside of the printhead, between the syringe and the bioink, and between the external surface of the printhead and the ambient air. The boundary condition could be described as follows:

(1) $$-n\cdot \left(-k\nabla T\right)=h(T_{ext}-T)$$

where n is the normal vector of the boundary, h is the heat transfer coefficient (W/(m^2^·K)), and $T_{ext}$ is the temperature of the surrounding medium (K).

The heat transfer coefficient inside the printhead could be assumed to be a constant value of 15 W/(m^2^·K). The bioink’s velocity was assumed constant, and its temperature decreased slightly.

The heat transfer coefficient with air was assumed to be a free-convection process and could be described as follows [20]:

(2) $$h=\frac{k}{L}f\left(\theta \right){Gr}^{1/4}$$

where k is the thermal conductivity of air, L is the typical length, and $f\left(\theta \right)$ is an empirical coefficient as a function of the incidence angle θ; Gr is the Grashof number.

In order to study both the temperature distribution and heat transfer velocity, static and transient simulations were performed in the thermal analysis. The parameters selected for the simulation can be seen in Table 1.

#### 2.5.2. Flow Analysis of Extrusion Process

To investigate how temperature distribution could affect the extrusion process, a flow analysis was conducted. Since the bioink was treated as an incompressible, laminar fluid, the momentum and continuity equations by COMSOL were as follows:

(3) $$\rho \frac{\partial u}{\partial t}+\rho \left(u\cdot \nabla \right)u=\nabla \cdot \left[-pI+\mu \left(\nabla u+{\left(\nabla u\right)}^{T}\right)-\frac{2}{3}\mu \left(T\right)\left(\nabla \cdot u\right)I\right]+\rho g+F_{st}$$

(4) ρ∇⋅u=0

where ρ is the density (kg/m^3^), u is the velocity vector (m/s), p is the pressure (Pa), μ is the dynamic viscosity (Pa·s), T is the absolute temperature (K), I is the unit tensor, and $F_{st}$ is the surface tension force (N/m^3^). In this study, the densities of both air and bioink were set as $1\times 10^{3}$ kg/m^3^.

The surface tension force can be calculated as follows:

(5) $$F_{st}=\sigma \delta \kappa n$$

where σ is the surface tension coefficient (N/m), δ is a Dirac delta function that is nonzero only at the fluid interface, κ is the curvature, and n is the interface normal. The surface tension coefficient was set to 0.7 N/m, and all other functions could be calculated as follows:

(6) κ=−∇⋅n

(7) $$n=\frac{\nabla \phi }{\left|\nabla \phi \right|}$$

(8) δ=6|ϕ(1−ϕ)||∇ϕ|

Since the bioink in this research was a shear-thinning fluid, it was described by the power law (Ostwald de Waele) model to demonstrate non-Newtonian flow behavior, as shown in Equation (10):

(9) $$\eta =\frac{\tau }{\dot{\gamma }}=K{\dot{\gamma }}^{n-1}$$

where η is the apparent viscosity (Pa·s), τ is the shear stress (Pa), K is the flow consistency factor (Pa·s^n^), $\dot{\gamma }$ is the shear rate (s^−1^), and n is the flow behavior index [21]. To find those values, Equation was transformed into logarithm form:

(10) $${log}_{10}\eta =(n-1){log}_{10}\dot{\gamma }+{log}_{10}K$$

K and n values were derived from rheological data by applying linear regression fitting with the logarithm data of related shear rate and viscosity. For shear-thinning fluid, the viscosity decreased with increased shear rate, which meant that n was less than 1.

In this study, two-phase flow was analyzed. To solve the two-phase flow, the level-set method was used, and the transport equation of the two-phase interface could be demonstrated as follows:

(11) $$\frac{\partial \phi }{\partial t}+u\cdot \nabla \phi =\gamma \nabla (\epsilon \nabla \phi +\phi \left(1-\phi \right)\frac{\nabla \phi }{\left|\nabla \phi \right|})$$

where ϕ is the level-set function of the gas–liquid interface, γ is the reinitialization parameter (m/s) used to solve the equation, ϵ is the controlling interface thickness (m), and u is the flow velocity (m/s).

In this study, the 0.5 contour of the level-set function defines the interface, where equals 0 in air and 1 in bioink. Since γ determined the amount of reinitialization, a suitable value of *γ* was the maximum magnitude occurring in the velocity field. We used $\epsilon =h_{c}/2$, where $h_{c}$ is the maximum mesh size.

In addition, since the density and viscosity were changing in the two-phase flow, the smoothing process was applied with the use of level-set function ϕ:

(12) $$\rho =\rho_{1}+(\rho_{2}-\rho_{1})\phi$$

(13) $$\mu =\mu_{1}+(\mu_{2}-\mu_{1})\phi$$

where ϕ is the level-set function, $\rho_{1}$ is the density of air, $\rho_{2}$ is the density of the bioink, $\mu_{1}$ is the viscosity of air, and $\mu_{2}$ is the viscosity of the bioink.

The geometrical features used for flow analysis were measured and calculated based on the homemade printhead with a 3 mL syringe, and the dimensions are listed in Table 2.

Boundary conditions are demonstrated in Figure 1. The inlet was on the top of the syringe and the flow rate was at a constant speed of 10 mm^3^/s, which derived from conventional extrusion flow rate. The outlet was the nozzle tip with 101,325 Pa, which was 1 atm. All other boundaries were considered to be under the “no slip” condition.

## 3. Results

### 3.1. Rheological Properties of the Bioink

**Figure 2:**
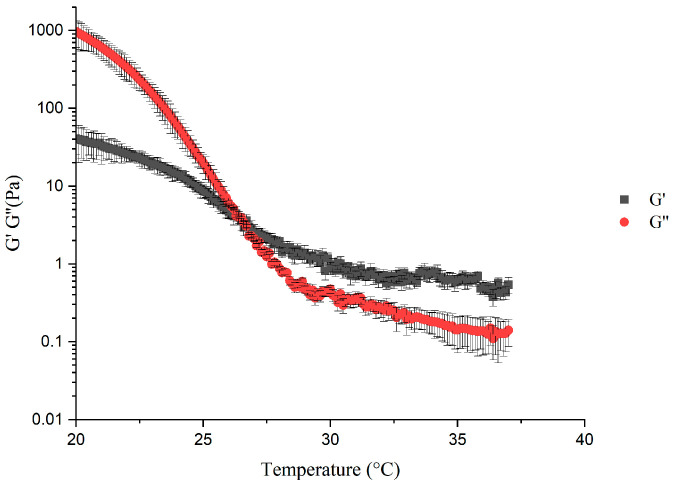
Rheological experiment to determine the gel point of the bioink by an oscillatory test.

The oscillatory test was conducted to demonstrate the viscoelasticity of the bioink, and it is a common method of determining the gel point. When the elasticity of the material is higher than the viscosity, that is, G′ > G″, the elasticity of the material plays a major role, as the material can be considered to be in a gel state; when the elasticity of the material is lower than the viscosity of the material, that is, G′ < G″, the material can be considered to be in a solution state. Therefore, the gel point of the bioink can be considered the intersection point of the storage modulus curve and the loss modulus curve, that is, G′ = G″ is the corresponding gel-point temperature value. The results are shown in Figure 2: when the temperature was higher than 26.1 °C, the bioink exhibited a solution-like state; when the temperature was below 26.1 °C, the bioink exhibited a gel-like state, which meant that the gel-point temperature of the bioink was 26.1 °C. Therefore, 26.1 °C served as a reference of proper gel-point temperature for the following studies. To investigate the viscosity parameter, an additional group of rotational experiments was conducted at 26.1 °C.

From the rotational test results, consistency factor K and flow behavior index n from the power law model were calculated by applying linear regression. The results are demonstrated in Table 3. The calculated fluid properties were used in the following flow analysis studies.

### 3.2. Finite Element Analysis of Printing Process

#### 3.2.1. Thermal Analysis Results

**Figure 3:**
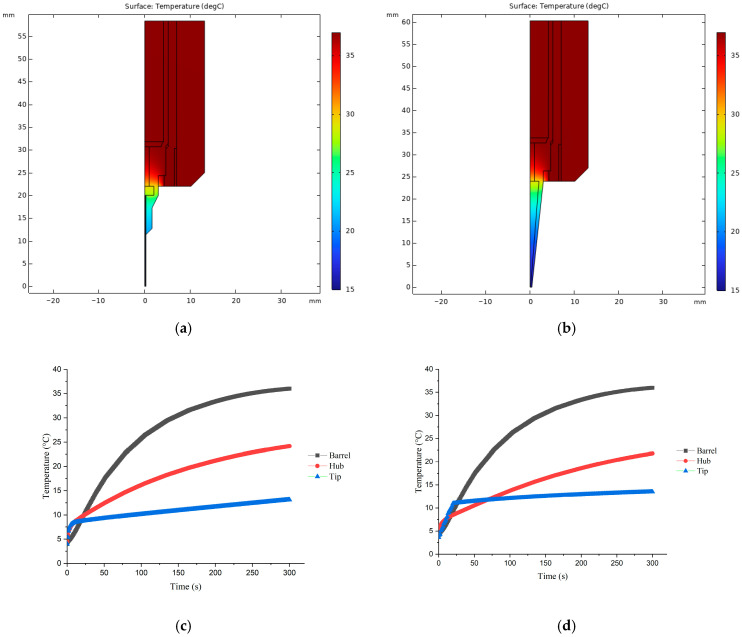
Static and transient thermal simulation results: (**a**) static thermal distribution with SS tip; (**b**) static thermal distribution with TT tip; (**c**) heat transfer velocity with SS tip; (**d**) heat transfer velocity with TT tip.

Figure 3 presents a detailed thermal analysis of the printhead, showcasing the temperature distribution within to illustrate heat transfer dynamics. The static study (Figure 3a,b) reveals the temperature gradient from the barrel to the nozzle tip, while the transient study (Figure 3c,d) demonstrates the velocity of bioink heat transfer at different parts. In configurations utilizing a stainless steel (SS) tip, the heat transition is depicted from the heating film (set at 37 °C) through the copper sleeve and syringe barrel, with the bioink within the barrel warming to 36.02 °C within 5 min and subsequently peaking at 36.91 °C after 10 min. However, temperatures at the syringe hub and nozzle tip, being more exposed to ambient conditions, were markedly lower, registering at 26.01 °C and 17.90 °C, respectively, indicating significant thermal loss. A similar thermal profile was observed with the tapered tip (TT) printhead, showing 36.03 °C at the barrel, 24.16 °C at the hub, and 13.15 °C at the nozzle tip.

To address bioink gelation, the heating film was adjusted to 26.1 °C, identified as the optimal gel point via rheological analysis. At this setting, bioink temperatures reached 26.07 °C at the barrel, 21.46 °C at the nozzle hub, 17.88 °C at the SS tip, and 15.94 °C at the TT nozzle tip, a range that risks premature gelation, leading to nozzle clogging for temperature-sensitive bioinks.

The observed temperature gradient is primarily attributed to natural convection towards the cooler ambient temperature (15 °C) prevalent during bioprinting. While increasing the printhead temperature could mitigate this issue, it is essential to recognize that bioink viscosity is highly temperature-dependent, with excessive heating risking overly low viscosity that compromises print resolution. Thus, optimizing the bioprinting process necessitates not just arbitrary temperature adjustments but also a strategic approach to minimizing the temperature gradient between the syringe barrel and nozzle tip to ensure consistent bioink properties and avoid clogging.

#### 3.2.2. Flow Analysis Results

Effect of temperature on morphology, pressure, and shear stress

The influence of temperature on the viscosity of temperature-sensitive bioinks, and subsequently on the extrusion bioprinting process, was rigorously analyzed using numerical simulations for gelatin–alginate bioink. These simulations were conducted at four distinct temperatures, 37 °C, 30 °C, 26.1 °C, and 15 °C, chosen specifically to investigate the bioink’s extrusion filament morphology across different states.

**Figure 4:**
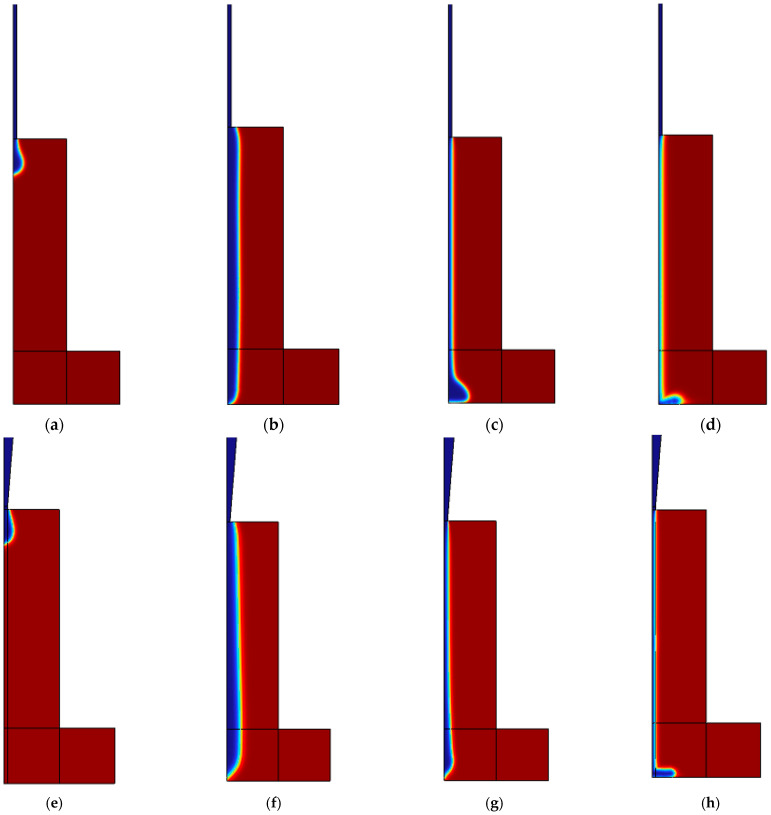
Filament morphologies from flow simulation for printhead with SS and TT tips at different temperatures: (**a**) SS tip at 37 °C; (**b**) SS tip at 30 °C; (**c**) SS tip at 26.1 °C; (**d**) SS tip at 15 °C; (**e**) TT tip at 37 °C; (**f**) TT tip at 30 °C; (**g**) TT tip at 26.1 °C; (**h**) TT tip at 15 °C.

The extrudability of the bioink is directly observable through its morphology at the nozzle tip, as depicted in Figure 4. The bioink exhibited varying level set functions under different thermal conditions, which served to delineate the bioink morphologies. Within this context, the 0.5 contour of the level set function was utilized to define the interface between the bioink (value of 1) and air (value of 0).

At 37 °C, the bioink’s low viscosity resulted in droplet formation rather than filament, indicating an under-gel state where surface tension predominated. Conversely, at temperatures of 30 °C, 26.1 °C, and 15 °C, filamentous structures were observed. For a printhead equipped with a stainless steel (SS) tip, the average filament diameters measured 808 µm at 30 °C, 288 µm at 26.1 °C, and 212 µm at 15 °C. With a tapered tip (TT) printhead, the diameters were slightly larger: 912 µm at 30 °C, 310 µm at 26.1 °C, and 236 µm at 15 °C. Since extrusion swelling is a typical phenomenon influenced by shear-thinning and viscoelasticity [22], the filament sizes were usually bigger than the diameter of the nozzle tip (250 µm), except for the over-gel state bioink when it was 15 °C.

**Figure 5:**
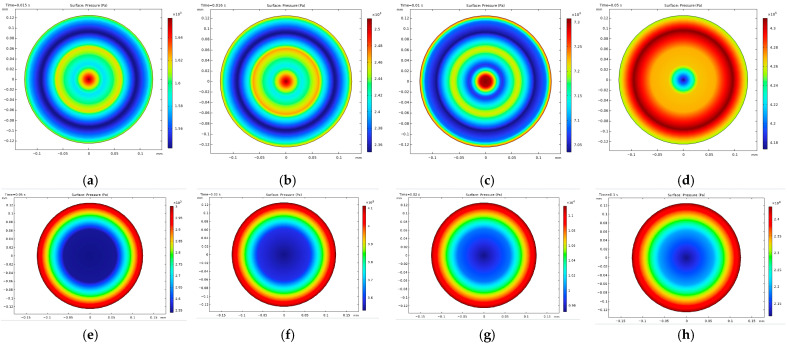
Pressure distribution at nozzle tip from flow simulation for printheads with SS and TT tips at different temperatures: (**a**) SS tip at 37 °C; (**b**) SS tip at 30 °C; (**c**) SS tip at 26.1 °C; (**d**) SS tip at 15 °C; (**e**) TT tip at 37 °C; (**f**) TT tip at 30 °C; (**g**) TT tip at 26.1 °C; (**h**) TT tip at 15 °C.

Pressure exerts a significant influence on the quality of bioprinting outcomes, with elevated pressures potentially leading to nozzle clogging and diminished cell viability [9]. As illustrated in Figure 5, the pressure dynamics vary notably with temperature changes and between nozzle types. For the SS tip, at 15 °C, pressure at the nozzle tip was recorded at 431 kPa, dropping significantly to 7.30 kPa at 26.1 °C and further to 2.51 kPa at 30 °C. In contrast, for the TT tip, pressure at the nozzle tip was measured at 24.3 kPa at 15 °C, decreasing to 11.1 kPa at 26.1 °C and 4.1 kPa at 30 °C.

The simulation data underscore the trade-off involved in achieving thinner filaments under over-gelation conditions, necessitating the application of considerably higher pressures to the bioink. This scenario is suboptimal for cell printing due to the detrimental effects of high pressure on cell viability. Additionally, we observed a pressure gradient within the TT nozzle tip, characterized by increasing pressure values from the tip center towards the wall. This gradient is attributable to the frictional forces exerted along the inner wall of the tip, highlighting the complex interplay between nozzle geometry, temperature, and pressure in the bioprinting process.

**Figure 6:**
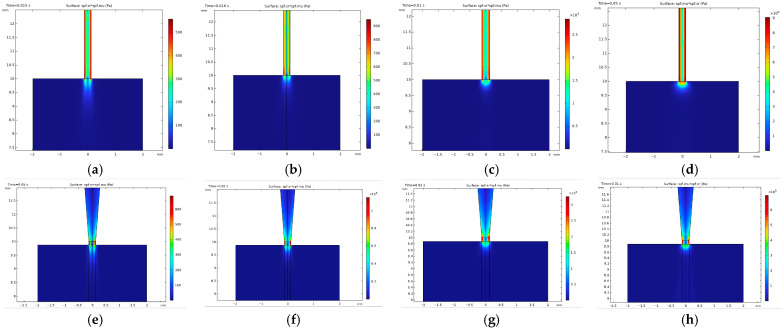
Shear stress distribution at nozzle tip from flow simulation for printheads with SS and TT tips at different temperatures: (**a**) SS tip at 37 °C; (**b**) SS tip at 30 °C; (**c**) SS tip at 26.1 °C; (**d**) SS tip at 15 °C; (**e**) TT tip at 37 °C; (**f**) TT tip at 30 °C; (**g**) TT tip at 26.1 °C; (**h**) TT tip at 15 °C.

In addition to pressure, shear stress was calculated and examined at the nozzle tip under different temperatures. Figure 6 showed that shear stress had the same trend as pressure: when it was 15 °C, the shear stress at the nozzle tip was 90 kPa for the SS tip and 6.748 kPa for the TT tip, and it reduced to 2.623 kPa (for the SS tip) and 3.216 kPa (for the TT tip) at 26.1 °C, 0.931 kPa (for SS tip) and 1.093 kPa (for TT tip) at 30 °C, and, eventually, 0.502 kPa (for SS tip) and 0.641 kPa (for TT tip) at 37 °C, which meant that cells experienced significantly lower shear stress as the temperature rose.

Notably, our analysis revealed that despite the maximum shear stress associated with SS tips being generally lower than that associated with TT tips, the distribution patterns provided insightful distinctions. Specifically, elevated stress levels were confined to the endpoints of TT tips, whereas bioink encountered consistent shear stress throughout its passage through SS tips. This pattern suggests that cells extruded using TT tips are subjected to relatively lower shear stress levels overall compared to those extruded through SS tips.

## 4. Discussion

In this investigation, we conducted a comprehensive analysis of the influence of temperature and nozzle types on the morphological characteristics of the filaments, as well as on the pressure and shear stress fields during the bioprinting process. Our flow analysis highlighted the critical role of bioink behavior at the nozzle, emphasizing the necessity of achieving an optimal gelation state to enhance the quality of bioprinted structures. However, our thermal assessments revealed the presence of a temperature gradient across the printhead, with a noticeable reduction in temperature at the nozzle tip. Given the profound effect of temperature on the fidelity of prints made with thermally sensitive bioinks, addressing this issue becomes imperative. To this end, we devised a hardware optimization strategy with the addition of thermal insulators on the printhead within this study.

**Figure 7:**
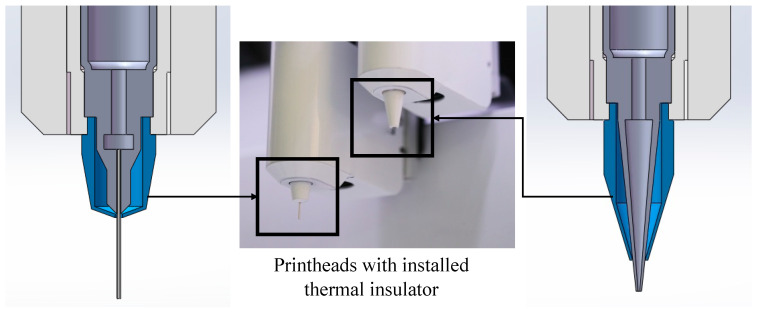
Thermal insulator design.

The design of thermal insulators for the nozzle tip (shown in Figure 7) was driven by the following considerations: Initially, simulations indicated the nozzle’s susceptibility to temperature fluctuations, which led to significant temperature gradients affecting the extrusion process. To counteract this, we employed copper for the insulator due to its excellent thermal conductivity, which facilitates the maintenance of a stable temperature at the nozzle tip. This modification ensured a uniform temperature distribution within the bioink, resulting in more consistent extrusion outcomes.

Furthermore, the standard practice of utilizing transparent materials for syringe nozzle tips poses a challenge when working with photo-sensitive bioinks, as it exposes the bioink to potential premature photo-crosslinking due to light exposure. By adopting opaque materials for the insulator tips, we mitigate this risk, thereby preserving the bioink’s integrity until the desired moment of crosslinking.

Additionally, the constrained spatial environment encountered when printing onto well plates necessitates careful consideration of the insulators’ geometries to avoid physical interference. To accommodate various nozzle configurations, we designed two distinct types of insulators tailored for tapered tips and for 1/2 inch stainless steel tips, respectively.

To rigorously assess the impact of thermal insulators on temperature management during the bioprinting process, we employed both thermal simulation and empirical printing experiments.

**Figure 8:**
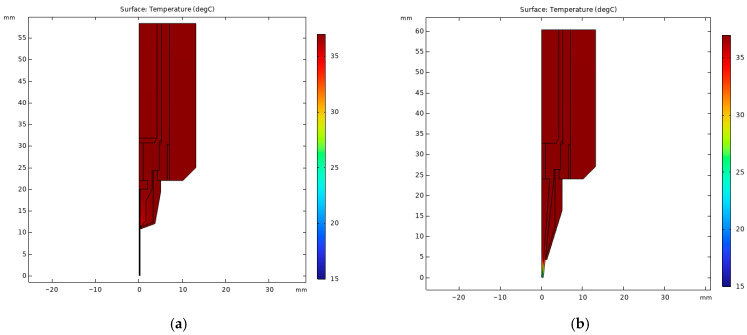

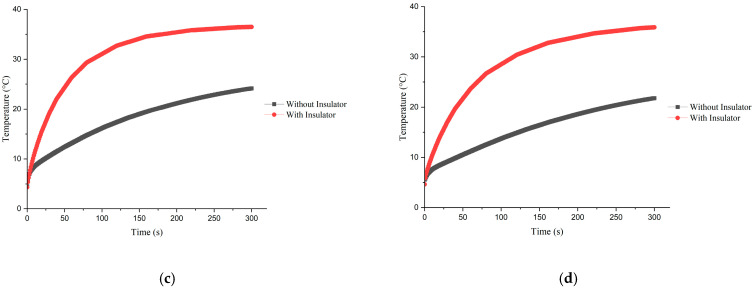
Static and transient thermal simulation results for printheads with thermal insulators: (**a**) static thermal distribution with SS tip; (**b**) static thermal distribution with TT tip; (**c**) heat transfer velocity at syringe hub with SS tip; (**d**) heat transfer velocity at syringe hub with TT tip.

The thermal simulation focused on analyzing the temperature distribution at the nozzle tip, with particular attention being paid to the efficacy of thermal insulation (Figure 8a,b). The results from the static study clearly showed that printheads equipped with thermal insulators exhibited superior thermal retention and a more uniform temperature distribution, effectively mitigating heat loss at the nozzle tip. Specifically, with the printhead temperature set at 37 °C, the temperature of the bioink at the hub reached 36.89 °C for the stainless steel (SS) tip and 36.98 °C for the tapered tip (TT), while average temperatures at the nozzle tip were observed at 36.88 °C (SS) and 35.60 °C (TT), and the lowest temperature of the tip could reach 31.02 °C (SS) and 28.77 °C (TT), respectively.

Moreover, the transient study, which aimed to evaluate the velocity of heat transfer, indicated that printheads fitted with thermal insulators not only enhanced thermal efficiency but also accelerated the heat transfer process (Figure 8c,d). This improvement could significantly reduce the operational time for users. The experimentally validated benefits of thermal insulators for maintaining optimal temperature distribution and expediting the heating process underscore their importance in achieving high-quality bioprinting outcomes, especially when working with temperature-sensitive bioinks.

Subsequent to our simulation efforts, empirical experiments were conducted to validate the simulation outcomes and assess the effectiveness of the optimized printhead design. However, it was difficult to measure the temperature of bioink directly, and the major purpose of the entire study was to achieve high fidelity structures with a stable printing process. Therefore, a porous cube model was fabricated under various thermal conditions using both traditional and modified printheads, with a particular focus on heating speed and the filament width as a critical measure of printing fidelity. For comparison with simulation results, the printing speed was set to 10 mm^3^/s

**Figure 9:**
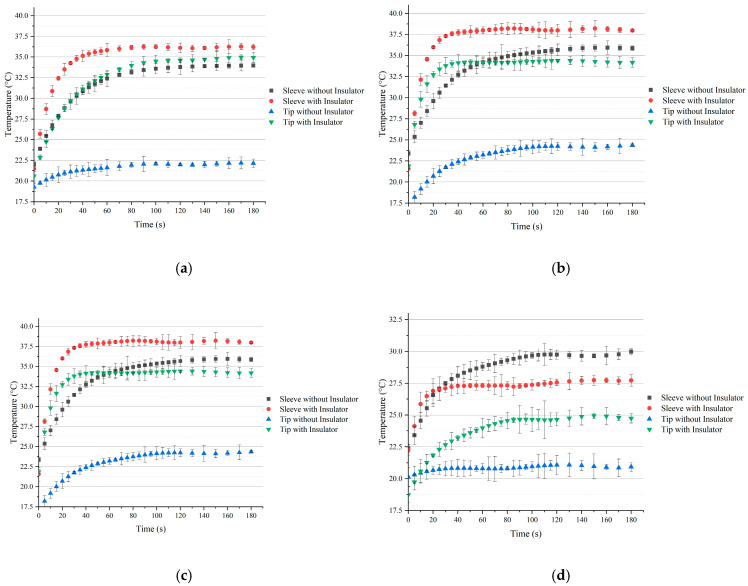
Temperature changes over time for printhead with and without thermal insulator at different heating targets: (**a**) heating target at 37 °C with SS tip; (**b**) heating target at 26.1 °C with SS tip; (**c**) heating target at 37 °C with TT tip; (**d**) heating target at 26.1 °C with TT tip.

Firstly, the heating speed was measured for a temperature of 37 °C and 26.1 °C via a two-temperature sensor (Pt100) attached at the heating sleeve near the heating film and the nozzle tip, respectively. The experiments lasted for 180 s (3 min), and values were collected every 5 s for the first 120 s and every 10 s for the last 60 s. The results are shown in Figure 9. It could be seen that for both types of tips, printheads with insulators were able to heat the printhead body, especially at the needle tip, to the desired target temperature. This result practically proved the conclusion of the thermal simulation results. Furthermore, it was noted that thermal insulator significantly improved the heating speed, as the printheads without insulators took about 120 s to heat up to the target temperature, whereas ones with insulators could approach the target temperature within 60 s. These test results showed that printheads with insulators were not only more accurate in terms of temperature control but also heated up faster. However, even with better-controlled thermal distribution and speed, in reality, it was difficult to precisely control the temperature within a range of 1 °C. It could be improved by applying more complex thermal control and stabler sensor installation.

**Figure 10:**
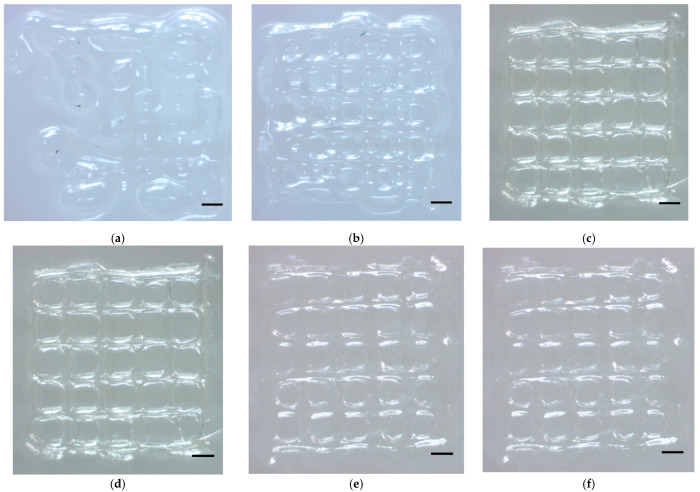
Extrusion bioprinting results at different temperature for printhead with SS tip with and without a thermal insulator (nozzle clogged at 26.1 and 15 °C when printing without insulator; thus, no corresponding results were demonstrated). Scale bar, 1000 µm: (**a**) 37 °C with insulator; (**b**) 30 °C with insulator; (**c**) 26.1 °C with insulator; (**d**) 15 °C with insulator; (**e**) 37 °C without insulator; (**f**) 30 °C without insulator.

Secondly, the experimental results, as illustrated in Figure 10, highlight the performance of a 250 µm 1/2 inch stainless steel (SS) nozzle with and without a thermal insulator (Figure 10a–d and Figure 10e,f, respectively). Morphologically speaking, when printing without an insulator at a temperature of 37 °C, the structure was successfully printed, with an average filament of 590 µm. However, when the temperature was set to 30 °C, the printed filaments exhibited over-gel conditions. The nozzles were even clogged when the temperature was set to 26.1 °C and lower due to the over-gel bioink and high viscosity.

In comparison, when printing with an insulator at 37 °C, the bioink remained in a solution state, proving inadequate for forming the intended structures due to insufficient gelation. Conversely, at a reduced temperature of 30 °C, the bioink was successfully extruded, albeit with a resolution yet to be satisfied, which was 765 µm on average. A further decrease in temperature to 15 °C induced an over-gel state, characterized by the formation of non-smooth filaments. Optimal printing results were observed when the printhead was set to 26.1 °C, where the filament width and the overall morphology—smooth and well structured with 338 µm—suggested that the bioink was extruded at its proper gelation temperature.

**Figure 11:**
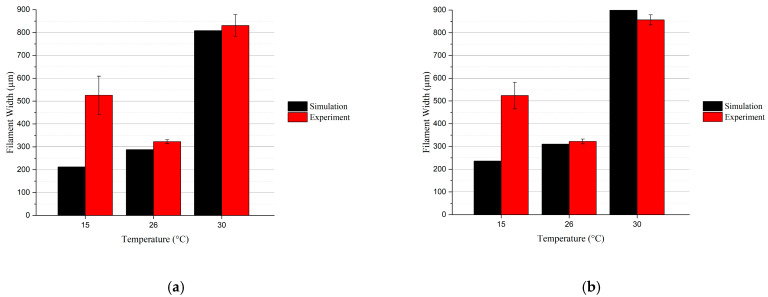
Average filament width at different temperatures from simulation and experiment results: (**a**) results for SS tip; (**b**) results for TT tip.

Moreover, Figure 11 demonstrated the average filament width of the simulation results and printing experiments with an insulator for the SS (Figure 11a) and TT tips (Figure 11b), respectively. The results showed similar trends for both tips. When the temperature was set to 26.1 °C and 30 °C, the filament widths from experiments were close to the simulation results. Notably, at a temperature of 15 °C, filaments from the experiment were significantly wider than the simulation results; the possible reason behind this result was the non-smooth filament with over-gel bioink. This condition not only facilitated the strict control of temperature by the printhead but also demonstrated the printhead’s capacity to maintain the bioink within ideal processing conditions for high-quality bioprinting outcomes.

In addition to printing with bioink, cell-laden 3D structures were also printed to investigate the cell viability with the optimized printhead. To examine the effects of cell printing results, the live/dead staining method was used to study the cell survival rate after printing, and the CCK-8 kit was applied to analyze cellular metabolic activity.

**Figure 12:**
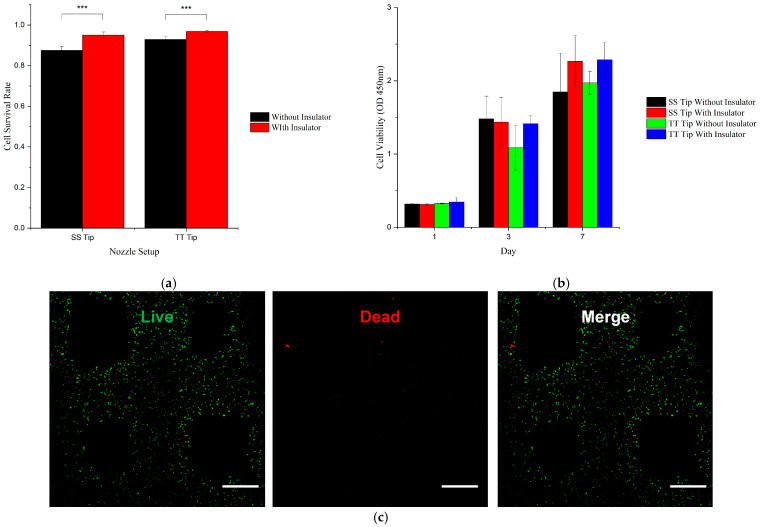
Cell viability analysis: (**a**) cell survival rate for SS and TT tips with and without insulator, where *** means *p* < 0.001, *t*-test; (**b**) cell proliferation for SS and TT tip with and without insulator; (**c**) live/dead staining of HUVEC-laden structure, where live cells are stained in green and dead cells are stained in red. Scale bar, 200 µm.

Figure 12a shows the cell survival rate calculated with live/dead staining by using different tips with and without an insulator. All groups showed relatively high cell viability (>85%), while printing without an insulator had relatively lower cell viability. Specifically, the SS tip without an insulator recorded 87.6% ± 1.9% viability, and it rose to 95.1% ± 1.5% when applying an insulator. The TT tip without an insulator had 92.9% ± 1.6% cell viability, and the TT tip with an insulator had 96.8% ± 0.6% cell viability. The results indicated that the printhead could deliver viable cells and the cell viability could improve when applying an insulator.

The cell proliferation data captured on days 1, 3, and 7, as presented in Figure 12b, indicate a consistent increase in CCK-8 signal across all groups, confirming the ongoing proliferation of HUVECs from day 1 to day 7. Notably, while all groups exhibited a growing trend over the seven days, groups with an insulator printhead demonstrated notably higher OD values by day 7 compared to those without an insulator. This suggests a more favorable environment for cell proliferation in the insulator groups. The prior simulations suggest that printing without an insulator is more susceptible to ambient temperature variations, potentially leading to increased shear stress at the nozzle tip. The integration of an insulator into the printhead not only enhances cell proliferation but also mitigates the negative impacts of environmental factors, making it a crucial component for improving bioprinting outcomes.

Overall, these findings broadly align with our simulation predictions, underscoring the insulator’s effectiveness in minimizing heat loss through natural convection at the nozzle tip and achieving a uniform temperature distribution across the printing zone.

## 5. Conclusions

The investigation conducted in this study has yielded valuable insights into the influence of temperature control and printhead design on the bioprinting process, particularly when working with temperature-sensitive bioinks like gelatin–alginate. By employing both FEM simulations and practical bioprinting experiments, we demonstrated the importance of maintaining a consistent temperature distribution across the printhead to prevent under- or over-gel status and ensure high-quality extrusion results. The introduction of thermal insulators emerged as a pivotal innovation, significantly reducing thermal gradients and enhancing the stability of bioink properties during bioprinting. These findings not only highlight the critical interplay between bioink rheology, temperature control, and mechanical forces within the bioprinting environment but also provide a solid foundation for future advancements in bioprinter design and operation. Ultimately, this research underscores the potential of precision-engineered bioprinting tools to revolutionize tissue engineering and regenerative medicine, offering promising avenues for the creation of complex, functional biological structures.

## Figures and Tables

**Table 1:** Material properties for simulation.

Material	Density (kg/m^3^)	Thermal Conductivity (W/(m·K))
Aluminum Alloy	2700	200
Copper	8960	400
Polypropylene	900	0.1
Air	1000	0.6
Bioink	1.225	0.025

**Table 2:** Geometrical features values for models with SS and TT tips.

Models with SS Tip	Value (mm)	Models with TT Tip	Value (mm)
Inner radius of barrel	4.15	Inner radius of barrel	4.15
Length of barrel	10	Length of barrel	10
Inner radius of syringe lock	1	Inner radius of syringe lock	1
Length of syringe lock	8.7	Length of syringe lock	8.7
Inner radius of syringe inlet	2	Inner radius of syringe inlet	2
Length of syringe inlet	2	Inner radius of nozzle	0.125
Inner radius of nozzle	0.125	Length of nozzle	24
Length of nozzle	20	Length of target pathway	10
Length of target pathway	10	Width of air pathway	2
Width of air pathway	2		

**Table 3:** Flow behavior index (n) and consistency factor (K) values calculated by linear regression at different temperatures.

Temperature (°C)	n	K (Pa·s)	R^2^
15	0.79	11.674	0.99
26.1	0.70	0.714	0.99
30	0.82	0.131	0.99
37	0.63	0.0863	0.99

## Data Availability

Data are contained within the article.
